# Are self-esteem and adult attachment affected by previous experiences of youth victimisation?

**DOI:** 10.1080/07853890.2021.1896148

**Published:** 2021-09-28

**Authors:** Catarina Teixeira, Catarina Santos, Rafaela Diogo, Telma Catarina Almeida

**Affiliations:** a IUEM – Instituto Universitário Egas Moniz, Caparica, Portugal; b LabPSI – Laboratório de Psicologia Egas Moniz, CiiEM – Centro de Investigação Interdisciplinar Egas Moniz, IUEM, Caparica, Portugal

## Abstract

**Introduction:**

Some research show the impact of the traumatic experiences of emotional abuse during childhood in an insecure attachment style [1], leading to a negative attitude towards oneself and towards others [2]. Studies also show that child abuse is a significant predictor of low self-esteem in adulthood [3]. The objectives of the current study are to analyse the relationship between the youth victimisation and self-esteem in adulthood, the adult attachment and the youth victimisation, and the self-esteem in adults and the adult attachment. This study is important to show, in a Portuguese sample, how these variables are linked, providing knowledge about the implications of previous experiences of youth victimisation.

**Materials and methods:**

The sample comprised 109 Portuguese participants, with ages between 18 and 68 years old (*M* = 33.96, SD = 13.97), and the majority was female (*n* = 82, 77.4%). Participants responded online to a sociodemographic questionnaire, a Childhood Trauma Questionnaire (CTQ) [4], a Rosenberg Self-Esteem Scale (RSES) [5], and an Adult Attachment Scale-R (AAS-R) [6]. Portuguese versions of the questionnaires were used.

**Results:**

The total score of the RSES revealed a significant statistical correlation with the CTQ subscales: Emotional Abuse (*r*=–0.233, *p*=.016), Emotional Neglect (*r*=–0.201, *p*=.039), and Physical Neglect *(r*=–0.235, *p*=.015). The total score of the CTQ showed significant statistical and correlations with the total score of the AAS-R and with its subscales: Anxiety (*r* = 0.198, *p*=.042), Close (*r* = 0.477, *p<.*001), and Depend (*r* = 0.445, *p*<.001). The factor Anxiety in the AAS-R showed a significant statistical correlation with the CTQ subscale Emotional Abuse (*r* = 0.349, *p*<.001). The subscale Close in the AAS-R showed significant statistical correlations with the CTQ subscales: Emotional Abuse (*r*=–0.266, *p*=.006), Emotional Neglect (*r*=–0.346, *p*<.001), Physical Neglect (*r*=–0.244, *p*=.012).The subscale Depend on the AAS-R revealed a significant statistical correlation with the CTQ subscales: Emotional Abuse (*r*=–0.249, *p*=.010). The RSES also showed a significant statistical correlation with the AAS-R subscales: Anxiety (*r*=–0.590, *p*<.001), Close (*r* = 0.511, *p*<.001), and Depend (*r* = 0.354, *p*<.001).

**Discussion and conclusions:**

This study highlights the relationship between experiences of youth victimisation and the increase of attachment anxiety in adulthood, decreased self-esteem, comfort with proximity, and confidence in others concerning the attachment in adults. The current study corroborates previous findings [1,2]. This research contributes to the practice of clinical and forensic psychology in the prevention and intervention in childhood trauma, showing, in a Portuguese sample, the impact of the social implications of trauma on attachment style, and self-esteem, during adulthood. However, although this research achieved important results, further studies are recommended, developing a theoretical model with those variables, and with a larger sample.
